# Mineral Composition of *Olea europaea* L. Leaves and Tisanes

**DOI:** 10.3390/plants14233566

**Published:** 2025-11-22

**Authors:** Aleksandra Pashtetskaia, Alexandra Kravtsova, Alexandra Peshkova, Inga Zinicovscaia, Oksana Shevchuk

**Affiliations:** 1 Federal State Funded Institution of Science, The Nikitsky Botanical Gardens–National Scientific Center of the Russian Academy of Sciences, Nikita, 298648 Yalta, Russia; 2 Joint Institute for Nuclear Research, 6 Joliot-Curie Str., 1419890 Dubna, Russia; 3 Department of Nuclear Physics, Horia Hulubei National Institute for R&D in Physics and Nuclear Engineering, 30 Reactorului Str. MG-6, 077125 Magurele, Romania

**Keywords:** *Olea europaea* L., leaves, tisanes, ICP-OES, therapeutic properties, mineral composition

## Abstract

The study aimed to identify mineral determinants of potential therapeutic effects of *Olea europaea* L. leaves from introduced cultivars of the Southern Coast of Crimea and to assess their suitability for functional aqueous tisanes. Using ICP-OES, eighteen macro- and micro-elements were identified in dried leaves, with K and Ca predominant (>10.0 g/kg), followed by P, S, and Mg (>1.0 g/kg). Maximum values occurred in ‘Nikitskaya’ (K 15.6 g/kg; S 2.05 g/kg; and P 1.97 g/kg) and ‘Tlemcen’ (Ca 18.6 g/kg and Mg 1.46 g/kg). Extractability into infusion (2 g/100 mL, 60 min) reached 325 mg/L for K, 26 mg/L for Ca, 48 mg/L for S, 18 mg/L for P, and 9 mg/L for Mg. Potentially toxic elements were below detection limits, indicating the safety of both the raw material and beverage. Principal component, correlation, and Ward clustering analyses highlighted ‘Nikitskaya’, ‘Lomashenskaya’, and ‘Coregiolo’ as having the highest cumulative mineral value among the tested six cultivars. Overall, the findings support the feasibility of olive-leaf tisanes as accessible sources of K, Ca, S, P, and Mg, with potential contributions to antioxidant defense, blood-pressure regulation, and lipid and carbohydrate metabolism.

## 1. Introduction

The European olive (*Olea europaea* L.) is one of the oldest cultivated plants, shaping the landscapes of the Mediterranean and Black Sea regions and playing a significant role in local agroecosystems and human nutrition [1]. In recent decades, interest in this species has grown considerably due to the expansion of its cultivation area and the use of not only its fruits and oil but also its leaves as a source of biologically active compounds [2,3].

Olive leaves have traditionally been used in folk medicine as raw material for the preparation of infusions and extracts with antioxidant, hypotensive, and anti-inflammatory properties [4,5], which is supported by modern evidence confirming the presence of polyphenols, flavonoids, and a wide range of mineral elements [6]. Recently, the importance of whole olive leaf and its extract is rapidly increasing in both the pharmaceutical and food industries—not only as a functional food product but also as an ingredient in food additives and preservatives [6,7].

The mineral composition of olive leaves, including their macro- and micro-element content, is determined by a complex of factors—the genotype of the cultivar, agricultural practices, soil composition, and the region’s climate [8,9]. Studies concerning the elemental composition of olive leaves and nutrient uptake by different varieties showed significant differences in the mineral composition of leaves even when they were grown under the same environmental conditions and agricultural practices [10,11,12,13,14,15].

The macro-elements (Ca, Mg, P, K, S), and micro-elements (Fe, Zn, Cu, Mn) are essential not only for the plant’s biological functions but also for the nutritional value and therapeutic properties of its processed products [16,17]. Infusions prepared from olive leaves represent a viable and affordable source of mineral substrates to address disorders related to essential elements such as Na, K, Mg, Ca, Mn, Fe, and Cu deficiencies [11]. According to Cavalheiro et al. [10] infusions prepared from olive leaves could allow 100% of the recommended daily intake of Fe and Cu.

The study of the mineral composition of *Olea europaea* leaves cultivated on the Southern Coast of Crimea is of particular importance. This region is characterized by a unique combination of sub-Mediterranean climate, high insolation, and specific soil conditions, which promote the accumulation of elements in plants [18,19]. Olives cultivated in the SCC have demonstrated their high nutritional and dietary value, which can reasonably be extrapolated to the leaves as a technologically accessible and sustainably available byproduct (agro-waste) with nutraceutical potential [20].

The present study aimed to provide a scientifically grounded characterization of the mineral profile of *Olea europaea* (*O. europaea)* leaves from six different cultivars grown under the same ecological conditions of the Southern Coast of Crimea (SCS). A set of six introduced cultivars (‘Nikitskaya’, ‘Tlemcen’, ‘Aglando’, ‘Coregiolo’, ‘Lomashenskaya’, ‘Tolgomskaya’) was purposefully assembled to obtain a representative cross-section of the genetic–geographic and morpho-typic diversity of olives stably cultivated on the SCC. The ‘Nikitskaya’ cultivar was selected from trees growing on the territory of the Nikitsky Botanical Garden (NBG) and is an original NBS cultivar; ‘Tlemcen’, ‘Aglando’, and ‘Coregiolo’ are introduced cultivars of Mediterranean origin; ‘Tolgomskaya’ and ‘Lomashenskaya’ are introduced cultivars of Caucasian origin (Black Sea coast of the Caucasus). All plants were obtained through the cutting method and represent vegetative clones, which ensure a high degree of genetic uniformity within each cultivar. All cultivars have been acclimatized for many years and are grown under identical soil and climatic conditions with a uniform natural agricultural background. This ensures the comparability of the sample and minimizes the influence of agricultural practices.

The objectives of the study were the following: (i) to determine the content of macro- and micro-elements in the leaves of six cultivars; (ii) to evaluate the extractability of elements into infusion; (iii) to apply different statistical approaches in order to discriminate cultivars based on their elemental composition. The investigation of the mineral composition of *O. europaea* leaves from the SCC has dual significance: (i) fundamental—for understanding plant physiology and elemental metabolism under sub-Mediterranean climate conditions; and (ii) applied—for the development of region-specific functional food products and the establishment of quality standards.

## 2. Results and Discussion

Previous studies on herbal teas from the Caucasus [21] and European plant materials [22,23] have shown that herbal infusions can provide up to 10–15% of the daily human requirement for certain macro- and micro-elements. These findings suggest that olive leaves grown in the SCC may represent a comparable or even more valuable source of mineral complexes. Analysis of the mineral profile of *O. europaea* leaves growing in the SCC allowed determination of 18 macro- and micro-elements (Table 1, full set of data is given in Table S1: leaves; Table S2: Tisanes). Among them, K and Ca predominated, with content exceeding 10.0 g/kg of dry weight. The group of elements with contents above 1.0 g/kg included P, S, and Mg. These findings are consistent with previous studies [11,20,24] on olive fruit-derived products, which likewise showed the dominance of K and Ca among macro-elements, and in agreement with literature data emphasizing the key roles of P and Ca in olive-leaf physiology [25,26]. The macro-elements—Ca, Mg, P, K, and S—provide structural and physiological functions, participate in the formation of cell walls, enzyme system activity, and maintenance of electrolyte balance [27].

Differences in element contents were observed among cultivars. Potassium, P, and S were most abundant in the ‘Nikitskaya’ cultivar, whereas Ca and Mg predominated in ‘Tlemcen’. Specifically, ‘Nikitskaya’ leaves were characterized by the highest content of K (15.6 g/kg), S (2.05 g/kg), and P (1.97 g/kg).

The highest Ca (18.6 g/kg) and Mg (1.46 g/kg) contents were determined in the leaves of ‘Tlemcen’. ‘Lomashenskaya’ and ‘Coregiolo’ leaves showed elevated levels of both macro- and micro-elements, making them promising candidates for use in functional food products. In ‘Tolgomskaya’ cultivar, moderate levels of elements were determined, but it demonstrated stability in its elemental composition. Among micro-elements, Fe, Zn, Mn, and Cu were predominant, serving as enzyme cofactors and contributing to the antioxidant properties of the leaves. Iron content ranged from 63 mg/kg (‘Tolgomskaya’) to 111 mg/kg (‘Tlemcen’). The highest Zn content was determined in ‘Lomashenskaya’ (21.3 mg/kg). Copper was present in concentrations ranging from 4.5 to 6.9 mg/kg, with the highest value in ‘Coregiolo’. These levels indicate the pharmacological potential of olive leaves as a source of essential trace elements. Among the group of conditionally essential elements, Sr is consistently detected in the infusion (approximately 0.10–0.15 mg/L), while Ni remains at the limit of quantification (<0.0004 mg/L). The concentrations of toxic and poorly studied components Ba, Pb, Cd, V, Al, and Cr in infusion were below ICP-OES detection limits. Collectively, this indicates selective extraction, a favorable safety profile, and the technological suitability of the raw material for producing infusions with functional mineral value.

**Figure 1:**
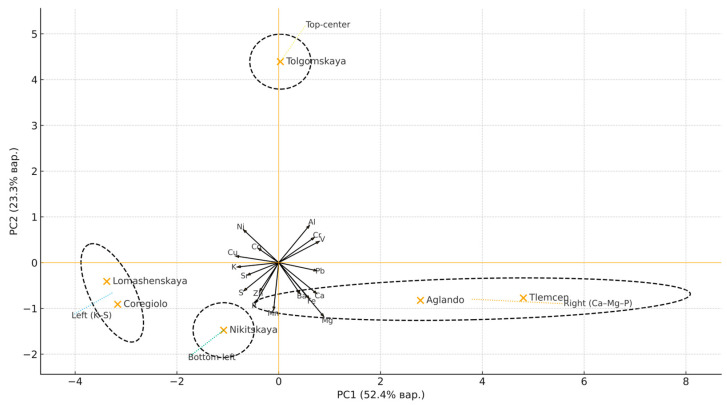
PCA biplot of the mineral composition of *Olea europaea* L. dry leaves (mg·kg^−1^).

The principal component analysis (PCA) of the distribution of varieties by element content (Figure 1) showed that the first two principal components explain 52.4% (PC1) and 23.3% (PC2) of the total variance.

In the PC1–PC2 projection, ‘Tlemcen’ and ‘Aglando’ (elevated Ca–Mg–P) are localized on the right. Moreover, ‘Tlemcen’ shows the highest Ca content among the varieties, and Ba and Fe are oriented along PC1 in the same direction (co-directed with Ca, P, and Mg), although its absolute levels remain low and are not the leading factors in separation (‘Aglando’ ≈ 2.7 mg/kg; ‘Tlemcen’ ≈ 2.6 mg/kg; maximum in ‘Nikitskaya’, 3.3 mg/kg; minimums in ‘Tolgomskaya’/‘Coregiolo’ ~1.6–1.4 mg/kg). ‘Lomashenskaya’ and ‘Coregiolo’ (high K/S) are located on the left, while ‘Nikitskaya’ is shifted to the lower left quadrant (K/S with low PC2) and is characterized by a combination of increased Sr (Ca analog) with relatively low Zn compared with other varieties. The Tolgomskaya variety is elevated in PC2 due to the contribution of the “aluminum” trace.

The cultivar, collecting period, and geographical location can greatly affect the content of macro- and micro-nutrients in olive leaves [28,29]. Since analyzed leaves were collected in the same period from the same geographical location, the cultivar can be considered the primary driver of variation in olive mineral content [30].

Taken together, this confirms that inter-varietal differentiation is determined by the balance of macro-element pools with the additional influence of lithogenic (soil–mineral) indicators—primarily Al and the associated V/Cr (±Pb) ratio. Therefore, for the technological selection of raw materials for a given mineral profile, it is rational to use axial markers (K, S) versus (Ca, Mg, P) and consider the lithogenic component as a modifier.

**Figure 2:**
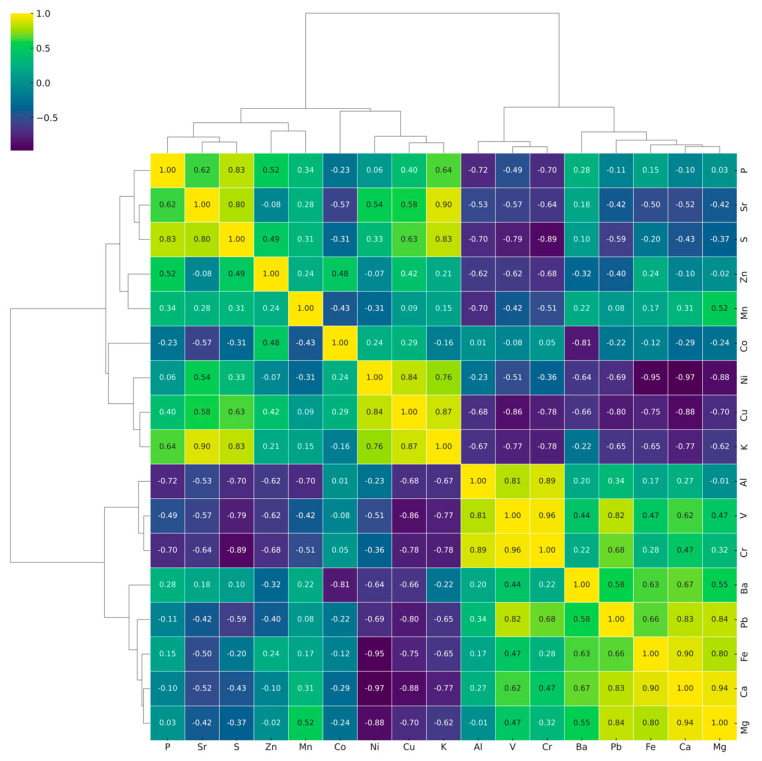
Clustered heatmap of standardized macro- and micro-element concentrations in leaves of six *Olea europaea* L. cultivars (positive correlations are colored warm, negative correlations are colored cool).

To verify the interpretations obtained from the PCA and to quantitatively assess the interrelationships between variables, pairwise dependency analysis was performed using Pearson correlation analysis, followed by the construction of a correlation heatmap (Figure 2). This provided a more detailed validation and enhanced the interpretative robustness of the results.

The heatmap reveals two groups of elements with strong correlations: a macro-element cluster (K–S–P–Mg–Ca, with Sr aligned) and an “aluminum” cluster (Al with V/Cr and partially Pb), with Al being anticorrelated with the macro-element block. Cu–Zn–Mn–Fe forms a moderately coherent micro-element contour. These relationships are fully consistent with the PCA results: PC1 describes the K–S ↔ Ca–Mg–P gradient, while PC2 reflects the contribution of the “aluminum” block. Thus, inter-cultivar differentiation is determined by the balance of macro-element pools, with a modifying role of the Al–V–Cr complex. For monitoring and raw material selection, representative markers (e.g., K/Ca and Al) are sufficient, avoiding collinear duplication of variables.

**Figure 3:**
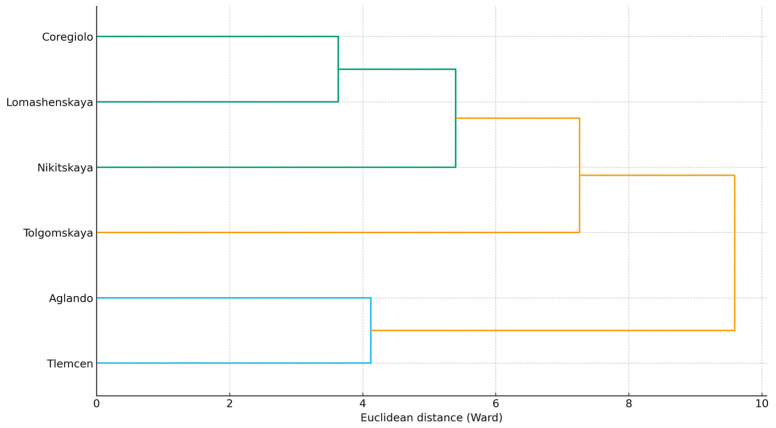
Hierarchical clustering (Ward’s method) of *Olea europaea* L. cultivars based on standardized concentrations of macro- and micro-elements in leaves (mg/kg): K–S–P cluster (‘Nikitskaya’, ‘Lomashenskaya’, ‘Coregiolo’), Ca–Mg–Fe–Ba cluster (‘Tlemcen’, ‘Aglando’), and a separate branch with an “aluminum” contribution (‘Tolgomskaya’).

Taking into account PCA and correlation analysis results, hierarchical clustering of the cultivars was applied to test the stability of groupings at the multivariate distance level (Figure 3). The Ward’s method, applied to standardized leaf data, minimizes within-cluster variance and allows verification of the stability of three clusters: the K–S–P cluster, the Ca–Mg–P cluster, and the “Al” cluster (Figure 3).

The dendrogram (Ward’s method; input—standardized content of macro- and micro-elements in leaves, mg/kg) shows the division of *Olea europaea* cultivars into three branches: a K-S-P cluster (‘Nikitskaya’, ‘Lomashenskaya’, ‘Coregiolo’), a Ca–Mg–Fe–Ba cluster (‘Tlemcen’, ‘Aglando’), and a separate branch with a pronounced Al contribution (‘Tolgomskaya’). This division fully supports the hypothesis of two dominant mineral composition modules. The pair ‘Tlemcen’–‘Aglando’ is united by elevated Ca–Mg–Ba–Fe content and relatively lower K/S (reflecting the PC1 gradient from PCA), while the trio ‘Nikitskaya’–‘Lomashenskaya’–‘Coregiolo’ gravitates toward a K-S-P profile. ‘Tolgomskaya’ separates due to the increased contribution of lithogenic (soil-derived) trace elements (Al ± V/Cr) and a moderate macro-element pool.

Such consistent differentiation shifts the inter-cultivar differences from the domain of structural typology into the realm of practical nutrition science. Therefore, an analysis was conducted comparing the inorganic profile (per kg of dry leaves) with the international dietary reference intakes (DRIs) and the ones established in Russia for key macro- and micro-elements (Table 2). This provides a rationale for the nutritional significance of *O. europaea* leaf-derived products, as well as for the determination of consumer metrics (serving size per day), standardization of raw material, and formulation of recipes accounting for technological extraction factors (leaf ↔ infusion). According to the obtained data (Table 2), the DRI values calculated using international and national norms were very close. The high extractability of major elements into the aqueous phase was observed: K ≈ 325 mg/L, Ca ≈ 26 mg/L, S ≈ 48 mg/L, P ≈ 18 mg/L, and Mg ≈ 9 mg/L, ensuring a physiologically meaningful dietary contribution [31,32,33,34].

These values are consistent with literature data on herbal teas from the Caucasus and Europe, where leaves and herbal mixtures also exhibited high levels of K and Mg [8,21,23]. Additionally, the extractability profile of conditionally essential and toxic/poorly studied elements demonstrated favorable selectivity and safety: Sr was consistently detected at ≈0.10–0.15 mg/L, whereas Ni, Pb, Cd, V, Cr, and Al were not transferred in the tisanes. Thus, the brewing process is characterized by selective extraction of nutritionally relevant macro-elements with minimal co-transfer of potentially toxic trace elements, thereby confirming the technological suitability of the raw material and ensuring a favorable toxicological profile of the resulting tisane.

**Figure 4:**
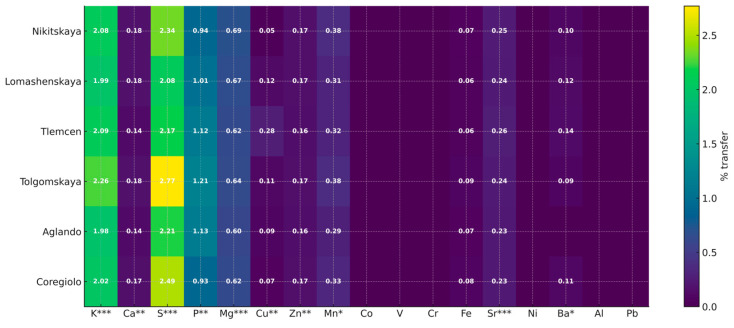
Heatmap of element transfer (%) from leaves to tisane (2 g per 100 mL, 60 min) across six *Olea europaea* L. cultivars. Significance: Asterisks denote inter-cultivar differences (ANOVA, Tukey HSD: * *p* < 0.05, ** *p* < 0.01, *** *p* < 0.001).

To visualize cultivar-specific extractability, a heatmap (Figure 4) of the “leaf → tisane” transfer ratios under standardized preparation conditions (2 g/100 mL, 60 min) was constructed. The diagram demonstrates a consistent dominance of S, K, and P, with moderate extractability of Mg and Ca, while most micro-elements show low transfer rates. Inter-cultivar differences manifest primarily in the amplitude of transfer (‘Tolgomskaya’—highest for S, K, P; ‘Nikitskaya’—for Mg, Zn, Mn; ‘Tlemcen’—for Cu, Sr, Ba), without altering the overall macro-element hierarchy. Thus, the heatmap can serve as an extraction passport, complementing concentration-based assessments and providing a rational basis for selecting raw material according to the desired mineral profile of the beverage.

The heatmap (Figure 4) reveals a stable macro-element core of extractability under the preparation conditions of 2 g/100 mL/60 min: the highest transfer ratios were observed for S (≈2.1–2.8%), K (≈2.0–2.3%), and P (≈0.93–1.21%), while Mg (≈0.60–0.69%) and Ca (≈0.14–0.18%) were extracted moderately. Micro-elements, in general, exhibited low extractability (tenths to hundredths of a percent), with near-zero values for Co, V, Cr, Ni, Al, and Pb. This indicates their minimal contribution to the mineralization of the aqueous extract and confirms the selectivity of water extraction toward major cations.

Inter-cultivar differences are amplitude-based without altering the macro-element hierarchy: ‘Tolgomskaya’ showed maxima for S, K, P and elevated Fe (up to 0.09%),—‘Nikitskaya’ had higher Mg, Zn, and Mn, and ‘Tlemcen’ stood out for Cu, Sr, and Ba. Thus, regardless of cultivar, the infusions are formed primarily by the K-S-P complex, which is of practical importance for recipe standardization and the targeted selection of raw material for beverages with specific mineral profiles.

Special attention was given to toxic elements (Cd, Pb, and Al); their concentrations in all samples were below detection limits. Furthermore, soil analyses of the production sites at the Nikitsky Botanical Garden–National Scientific Center (NBS-NSC) confirmed that the levels of bioavailable heavy metal forms in *O. europaea* products did not exceed maximum permissible concentrations, eliminating the risk of raw material contamination with toxic elements [20,24]. This minimizes regulatory risks in the development of functional beverages (tisanes) based on olive leaves and confirms the ecological safety and suitability of the studied cultivars for food and medicinal applications.

From a clinical and nutraceutical standpoint, chronic Ca deficiency (e.g., in osteoporosis) is traditionally compensated by diet and Ca supplements combined with vitamin D and/or magnesium. However, increased intake of Ca and vitamin D requires adequate magnesium levels, as Mg deficiency may lead to hypocalcemia and hypokalemia. A lack of inorganic phosphate (Pi) and/or energy substrates (ATP) is associated with musculoskeletal and neuromuscular dysfunctions, emphasizing the importance of P [38,39].

Manganese participates in the activity of mitochondrial Mn-SOD; although pronounced Mn deficiency is rare, maintaining sufficient levels is essential for antioxidant status [40]. The balance of Fe and Cu is critical for hematopoiesis and tissue respiration; Fe imbalance can cause anemia, while Cu metabolism disorders are linked to metabolic and neurodegenerative risks [6]. According to our data, the mineral profile (per kg of dry leaves) provides a substantial contribution to the recommended daily intake (DRI) levels of Ca, K, Mg, and P, as well as the micro-elements Fe, Zn, Cu, and Mn. Among the cultivars, ‘Nikitskaya’ shows the highest contribution of K and ‘Tlemcen’ the highest Ca levels. These results confirm the nutritional relevance of macro-elements (K, Mg, Ca, P) and transition metals (Mn, Fe, Cu, Zn), which are functionally involved in antioxidant defense and key enzymatic systems. Thus, aqueous infusions (tisanes) of olive leaves can be considered a source of mineral complexes with pronounced preventive potential.

A comparative analysis of olive cultivars based on the mineral profile of dried leaves reveals specific directions for their practical application. The ‘Tlemcen’ cultivar is characterized by a high content of Ca and Mg, making it promising for the prevention and correction of conditions associated with hypocalcemia and hypomagnesemia. The ‘Nikitskaya’ cultivar shows elevated levels of K, P, and S, indicating its relevance for addressing K deficiency and for use in programs aimed at supporting energy metabolism and antioxidant balance. The ‘Lomashenskaya’ and ‘Coregiolo’ cultivars demonstrate a higher total content of macro-elements and can be considered universal sources of mineral substrates, especially when combined with the polyphenolic components of the leaves.

Obtained results and the literature data indicate that *O. europaea* leaves grown on the Southern Coast of Crimea are not only a rich source of essential minerals but also possess significant therapeutic potential when consumed as herbal infusions (tisanes). This is consistent with international studies, confirming the health benefits of *O. europaea* leaves and herbal teas as components of preventive nutrition [4,6,20,21].

## 3. Materials and Methods

### 3.1. Olive Leaves Collection and Analysis

The studies were conducted on six *Olea europaea* cultivars: ‘Nikitskaya’, ‘Tlemcen’, ‘Aglando’, ‘Coregiolo’, ‘Lomashenskaya’, and ‘Tolgomskaya’. These cultivars were introduced and are cultivated under the conditions of the Southern Coast of Crimea (SCC), characterized by a sub-Mediterranean climate, high levels of insolation, and specific soil–geochemical conditions. Leaves sampling was conducted in the first ten days of April from trees aged 65–70 years. The trees are grown under identical soil and climatic conditions on a single experimental-production plot of the Nikitsky Botanical Garden under a natural agricultural background. The experimental olive orchard was established with a planting scheme of 5 m × 5 m, corresponding to a tree density of approximately 400 trees ha^−1^. The experimental-production plot of the Nikitsky Botanical Garden is located on the Southern Coast of Crimea (44°30′24″ N, 34°14′10″ E) in a moderately dry subtropical climate of the Mediterranean type, with a predominance of autumn–winter precipitation. The average annual temperature is +12 °C to +15 °C, the absolute minimum in winter is −13 °C, and the absolute maximum in summer is +39 °C. The amount of precipitation is 620–730 mm [41].

The plot is located on the limestone–dolomite bedrock of the SCC; the soil is represented by calcareous, weak soddy/brown forest soils with stoniness and thin eluvial–diluvial deposits. This results in high Ca and Mg carbonate content. According to Kostenko and Dunaevskaya [42], the agro-biocenoses of the Nikitsky Botanical Garden are characterized by moderate availability of Fe, Mn, Zn, and Cu, with low mobility of Al in the carbonate environment; Sr naturally accompanies Ca. Trace/Low-mobility forms are characteristic for V, Cr, Ni, and Co. The technogenic load, based on literature data [43], is low. Mineral fertilizers, pesticides, growth stimulants, and irrigation were not applied.

The leaves were collected from three model trees per cultivar. The leaves were air-dried to constant weight and then additionally dried at 105 °C for 24 h in a drying oven to remove residual moisture.

For analysis using a high-resolution inductively coupled plasma optical emission spectrometer, PlasmaQuant PQ 9000 Elite (Analytik Jena, Jena, Germany), the samples were brought into solution form. Approximately 0.3 g of dry material was weighed and placed in Teflon vessels, to which 3 mL of HNO_3_ (Honeywell Fluka 69% Suprapur, Sigma-Aldrich, Darmstadt, Germany) and 1 mL of H_2_O_2_ (Sigma-Aldrich 30% EMSURE, Darmstadt, Germany) were added. The vessels were left at room temperature for 30 min to remove volatile compounds. After this pre-treatment, the samples were subjected to microwave digestion in a MARS 6 system (CEM, Matthews, Pittsburgh, PA, USA). The program included temperature ramp-up–20 min, holding at 180 °C–15 min, and cooling–20 min. The resulting digests were diluted with deionized water to a final volume of 10 mL. Calibration was performed using a standard solution IV-STOCK-13 (Inorganic Ventures, Christiansburg, VA, USA), diluted in 2% HNO_3_ to concentrations ranging from 0.001 to 1 mg/L. All samples were analyzed in triplicates.

For quality control, the NIST 1575a (trace elements in pine needles) and NIST 1547 (peach leaves) reference materials were used. The recovery of elements ranged from 72 to 118%, which complied with international standards for analytical quality control (Table 3).

### 3.2. Tisane’s Preparation and Analysis

To prepare the tisanes, according to ISO 3103 [44] 2 g of dry, crushed (0.5–1.0 cm) olive leaves were poured into 100 mL of boiled (100 °C) filtered water and infused for 60 min at 80 °C. According to Vuong et al. [45], Długaszek and Mierczyk [46], and Durmus et al. [47], a brewing temperature of 80–100 °C and time of 60 min ensure high extraction of phenolic compounds and mineral components in the tisanes.

The resulting infusions were filtered using ashless filters and acidified with concentrated HNO_3_ (Honeywell Fluka 69% Suprapur, Sigma-Aldrich, Darmstadt, Germany). The ICP multi-element standard solution X (Producer: Merck KGaA, Darmstadt, Germany) was used for quality control of measurements. Single-element standards (Inorganic Ventures, Christiansburg, VA, USA) were used for Al, P, and S. The reproducibility of elemental values ranged from 86 to 116% (Table 4).

### 3.3. Statistical Data Analysis

For statistical data processing, analysis of variance (one-way and multifactor ANOVA) was used, followed by Tukey’s HSD post hoc test for multiple comparisons. Significant differences were considered at *p* < 0.05 (and *p* < 0.01 was additionally noted for key parameters). To identify hidden structures and groupings, principal component analysis (PCA), correlation analysis, and hierarchical clustering (using Ward’s method with Euclidean distance) were performed. All calculations were performed in Microsoft Excel and Statistica 14.0.1.

## 4. Conclusions

Conducted research demonstrated that leaves of *Olea europaea* cultivars grown on the Southern Coast of Crimea are a valuable source of macro- and micro-elements as shown by the ICP-OES results. High extraction of K, Ca, Mg, Mn, Zn, and S tisanes allow their use as an additional dietary source of important elements, while low level of potentially toxic elements in leaves and their insignificant transfer in tisanes confirms the environmental safety of plant material, supporting the conclusion that *O. europaea* leaves grown in the natural and climatic conditions of southern Crimea are not only effective but also safe phytotherapeutic raw materials. The principal component analysis, correlation, and clustering analyses present a coherent picture: inter-cultivar differentiation is governed by the balance between K and S versus Ca, Mg, and P (PC1: K–S ↔ Ca–Mg–P), with a modifying role of the “aluminum” complex (PC2: Al ± V/Cr), which can serve as a basis for standardized selection of raw material targeting a specific mineral profile.

It should be noted that this study has not conducted sensory evaluation on the tea infusion. Future studies are recommended to further conduct sensory evaluation of the tea infusion and test the potential link of the tea infusion with health benefits to confirm its quality and health association.

## Figures and Tables

**Table 1:** The content of major and minor elements in leaves and tisanes of *Olea europaea* L.

Element													*p*-Value	
	‘Nikitskaya’		‘Tlemcen’		‘Aglando’		‘Coregiolo’		‘Lomashenskaya’		‘Tolgomskaya’		Leaf	Tisane
	Leaf	Tisane	Leaf	Tisane	Leaf	Tisane	Leaf	Tisane	Leaf	Tisane	Leaf	Tisane		
Macro-elements, g/kg for leaves and mg/L for tisanes														
K	15.6 ^a^	325 ^a^	11.7 ^c^	244 ^c^	12.4 ^c^	244 ^c^	15.4 ^a^	310 ^a^^b^	14.9 ^a^^b^	298 ^b^	13.9 ^b^	314 ^a^^b^	<0.001	<0.001
Ca	14.8 ^b^	26 ^a^	18.6 ^a^	26 ^a^	17.2 ^b^	23 ^a^^b^	13.1 ^b^^c^	22 ^a^^b^	12.7 ^c^	22 ^b^	11.8 ^c^	21 ^b^	<0.001	0.004
P	1.97 ^a^	18 ^b^	1.82 ^b^^c^	20 ^a^	1.73 ^b^^c^	20 ^a^^b^	1.87 ^a^^b^	17 ^b^	1.92 ^a^^b^	19 ^a^^b^	1.7 ^c^	21 ^a^	<0.001	0.006
S	2.05 ^a^	48 ^a^	1.73 ^b^	37 ^c^	1.83 ^b^	40 ^b^^c^	1.94 ^a^^b^	48 ^a^	2.05 ^a^	43 ^b^	1.75 ^b^	49 ^a^	<0.001	<0.001
Mg	1.3 ^b^	8.9 ^a^	1.46 ^a^	8.9 ^a^	1.36 ^a^^b^	8.2 ^b^	1.29 ^b^	7.9 ^b^	1.18 ^c^	7.9 ^b^	1.14 ^c^	7.3 ^c^	<0.001	<0.001
Micro-elements														
Essential, mg/kg for leaves and mg/L for tisanes														
Cu	5.7 ^b^	0.001 ^b^	4.5 ^c^	0.01 ^a^	4.8 ^c^	0.001 ^b^	6.9 ^a^	0.01 ^b^	6.6 ^b^	0.01 ^a^	5.90 ^b^	0.01 ^b^	0.002	0.002
Zn	17.8 ^b^	0.03 ^b^	18.9 ^a^^b^	0.03 ^b^	18.3 ^a^^b^	0.03 ^b^	19.4 ^a^^b^	0.03 ^a^^b^	21.3 ^a^	0.04 ^a^	16.9 ^b^	0.03 ^b^	0.001	0.008
Mn	21.3 ^a^^b^	0.08 ^a^	20.7 ^a^^b^	0.07 ^b^	21.8 ^a^^b^	0.06 ^b^	22.4 ^a^	0.07 ^a^^b^	20.5 ^b^	0.06 ^b^	19.3 ^b^	0.07 ^a^^b^	0.012	0.012
Co	0.02 ^b^	<DL	0.03 ^a^^b^	<DL	0.01 ^b^	<DL	0.03 ^a^^b^	<DL	0.04 ^a^	<DL	0.03 ^a^^b^	<DL	0.008	-
V	0.18 ^b^	<DL	0.26 ^a^	<DL	0.21 ^a^^b^	<DL	0.15 ^b^	<DL	0.15 ^b^	<DL	0.23 ^a^^b^	<DL	0.004	-
Cr	0.12 ^a^	<DL	0.14 ^a^	<DL	0.12 ^a^	<DL	0.08 ^b^	<DL	0.08 ^b^	<DL	0.14 ^a^	<DL	0.005	-
Fe	86.0 ^b^	0.06 ^a^	111 ^a^	0.07 ^a^	95.1 ^a^^b^	0.06 ^a^	69.7 ^b^^c^	0.06 ^a^	86.7 ^b^	0.05 ^a^	63.2 ^c^	0.06 ^a^	<0.001	NS
Conditionally essential, mg/kg for leaves and mg/L for tisanes														
Sr	61.6 ^a^	0.15 ^a^	39.2 ^c^	0.10 ^c^	45.9 ^c^	0.11 ^b^^c^	53.9 ^b^	0.12 ^b^	49.9 ^b^^c^	0.12 ^b^	48.1 ^b^^c^	0.12 ^b^	<0.001	<0.001
Ni	0.51 ^a^^b^	<DL	0.38 ^b^	<DL	0.40 ^b^	<DL	0.55 ^a^^b^	<DL	0.52 ^a^^b^	<DL	0.59 ^a^	<DL	0.006	-
Toxic and poorly studied, mg/kg for leaves and mg/L for tisanes														
Ba	3.3 ^a^	<DL	2.6 ^a^^b^	0.004 ^a^	2.7 ^a^^b^	0.003 ^a^	1.4 ^b^	0.002 ^a^^b^	1.7 ^b^	0.002 ^a^^b^	1.5 ^b^	0.001 ^b^	0.003	0.015
Al	119 ^b^	<DL	146 ^b^	<DL	146 ^b^	<DL	80.4 ^c^	<DL	111 ^b^	<DL	168 ^a^	<DL	<0.001	-
Pb	0.36 ^a^	<DL	0.49 ^a^	<DL	0.38 ^a^	<DL	0.36 ^a^	<DL	0.30 ^a^	<DL	0.36 ^a^	<DL	NS	-
Cd	<DL	<DL	<DL	<DL	<DL	<DL	<DL	<DL	<DL	<DL	<DL	<DL	-	-

DL—detection limit, “^a^”—statistically significant highest values; “^b^”—intermediate values; “^c^”—statistically significant lowest values; letter combinations (^a^^b^, ^b^^c^)—values statistically not different from both adjacent groups; NS—not statistically significant (*p* > 0.05).

**Table 2:** The extractability of major and minor elements from leaves *Olea europaea* L. in tisanes (calculated for 200 mL).

Element	Daily Intake Norms (DRI) Russia [24]	Contribution of the Extract, % DRI	Daily Intake Norms (DRI) International	Contribution of the Extract, % DRI
K	1–2 g	3–7	3510 mg/day [35]	1.4–1.8
Ca	800–1200 mg	0.4–0.7	1000 mg/day [36]	0.42–0.52
P	400–1200 mg	0.3–0.9	700 mg/day [36]	0.49–0.60
S	500–1000 mg	1–2	n/a	n/a
Mg	300–400 mg	0.5–0.6	400/320 mg/day [36]	0.35–0.56
Cu	1–2 mg	0.20	0.9 mg/day [37]	0.22
Zn	10–20 mg	0.03–0.08	11/8 mg/day [37]	0.05–0.1
Mn	2–5 mg	0.24–0.8	2.3/1.8 mg/day [37]	0.52–0.89
V	20–30 mcg	<1.1–1.7	n/a	n/a
Cr	-	-	35/25 μg/day [37]	0.17–0.24
Fe	10–20 mg	0.05–0.14	8/18 mg/day [37]	0.06–0.18

n/a—not available.

**Table 3:** Quality control of ICP-OES measurements.

Element	SRM	Measured Values, mg/kg	Certified Values, mg/kg	Recovery, %
Al	1547	252 ± 0.03	249 ± 6.5	101
	1575a	579 ± 1.1	580 ± 30	100
Ba	1547	122 ± 1.55	124 ± 5.5	99
	1575a	4.92 ± 0.01	6.0 ± 0.2	82
Pb	1547	0.91 ± 0.0002	0.87 ± 0.02	104
Zn	1547	18.18 ± 0.05	18.0 ± 0.53	102
	1575a	37.5 ± 0.16	38 ± 2	99
V	1547	0.33 ± 0.01	0.37 ± 0.04	88
Mn	1547	97.8 ± 0.16	97.8 ± 1.8	100
Cr	1547	0.72 ± 0.02	1 *	72
Ni	1547	0.62 ± 0.001	0.69 ± 0.10	90
Sr	1547	61.7 ± 0.38	53.0 ± 5.0	116
P	1547	1525 ± 7	1371 ± 82	111
	1575a	1157 ± 5.1	1070 ± 80	108
Ca	1575a	2620 ± 2.25	2500 ± 100	105
Mg	1575a	1031 ± 0.27	1060 ± 170 *	97
S	1547	1634 ± 15	2000 *	82
Fe	1547	204 ± 3	220 ± 6.8	94
K	1575a	4391 ± 2.33	4170 ± 70	105

* Informative values.

**Table 4:** Quality control of the ICP-OES used in the present study.

Element	Measured Values, mg/L	Certified Values, mg/L	Recoveries, %	Detection Limit, mg/L
Al	0.05 ± 0.0001	0.05	99	0.004
Ba	0.04 ± 0.0001	0.05	86	0.003
Cd	0.02 ± 0.0007	0.02	116	0.0003
Co	0.03 ± 0.0001	0.03	112	0.0004
Zn	0.05 ± 0.0004	0.05	101	0.009
V	0.05 ± 0.0007	0.05	100	0.0017
Mn	0.03 ± 0.0001	0.03	91	0.006
Cr	0.02 ± 0.0006	0.02	106	0.0003
Ni	0.05 ± 0.0001	0.05	96	0.0004
Sr	0.11 ± 0.0001	0.10	110	0.003
Fe	0.10 ± 0.0003	0.10	103	0.002
Ca	37.4 ± 0.28	35.0	107	0.43
Mg	13.1 ± 0.05	15.0	87	0.07
K	3.26 ± 0.02	3.00	109	0.02
Cu	0.02 ± 0.0001	0.02	105	0.0004
Pb	0.03 ± 0.0008	0.03	98	0.0003

## Data Availability

The original contributions presented in the study are included in the article/Supplementary Material, further inquiries can be directed to the corresponding author.

## Supplementary Materials

The following supporting information can be downloaded at: https://www.mdpi.com/article/10.3390/plants14233566/s1, Table S1: leaves; Table S2: Tisanes.
